# Microbiological characteristics and risk factors on prognosis associated with *Acinetobacter baumannii* bacteremia in general hospital: A single-center retrospective study

**DOI:** 10.3389/fmicb.2022.1051364

**Published:** 2022-11-10

**Authors:** Zhiyong Wei, Shuai Zhou, Ying Zhang, Lin Zheng, Lina Zhao, Yan Cui, Keliang Xie

**Affiliations:** ^1^Department of Critical Care Medicine, Tianjin Medical University General Hospital, Tianjin, China; ^2^Department of Pathogen Biology, School of Basic Medical Sciences, Tianjin Medical University, Tianjin, China; ^3^Department of Anesthesiology, Tianjin Institute of Anesthesiology, Tianjin Medical University General Hospital, Tianjin, China

**Keywords:** *Acinetobacter baumannii*, bacteremia, outcome, risk factors, SOFA score, APACHE II score, MDR—(multi drug resistant)

## Abstract

**Objective:**

*Acinetobacter baumannii* is one of the most important pathogenic bacteria causing nosocomial infections and has a high mortality rate. Assessment of the microbiological characteristics and risk factors on prognosis associated with *A.baumannii* is essential. In this study, we aimed to investigate the clinical characteristics and prognostic risk factors of patients with *A.baumannii* bacteremia.

**Patients and Methods:**

This study retrospectively analyzed the antibiotic resistance of pathogens based on the clinical data of *A.baumannii* bacteremia patients presented in a tertiary teaching hospital from 2017 to 2022. Logistic regression and decision tree identified the prognostic risk factors for patients with baumannemia. Kaplan-Meier method was used for survival analysis between MDR and Non-MDR groups. The area under receiver-operating characteristic curve (ROC curve) was used to compare the predictive value of the APACHE II score and Sequential Organ Failure Assessment (SOFA) score.

**Results:**

A total of 110 patients with positive *A. Baumannii* blood cultures were included. Most of the patients were from intensive care unit (ICU) wards. The drug sensitivity results showed that the resistance rate of *A. baumannii* to colistin was the lowest (1.1%), followed by tigecycline (3.6%).

The survival time of MDR group was significantly shorter than that of Non-MDR group. Multivariate analysis showed that, APACHE II score and SOFA score were independent risk factors affecting the prognosis of 28 days of *A.baumannii* bacteremia. And both scores displayed excellent AUROCs (SOFA: 0.909, APACHE II: 0.895 in predicting 28-day mortality). The two scoring systems were highly correlated and predicted no significant difference (*r*^2^ = 0.4410, *P* < 0.001). We found that SOFA > 7 and APACHE II > 21 are associated with significantly higher mortality rates.

**Conclusion:**

*A.baumannii* bacteremia have the highest incidence in the ICU, with high drug resistance and mortality rates. The survival time of patients with MDR *A. Baumannii* bacteremia was significantly shortened. The SOFA score and APACHE II score can reflect the severity of *A.baumannii* bacteremia patients and evaluate the 28-day prognosis. In addition, for the convenience of calculation, the SOFA score may be more clinically useful than the APACHE II score in predicting the mortality rate of *A.baumannii* bacteremia.

## Introduction

Bacteremias are life-threatening conditions affecting patients in the hospital. The timely and effective application of antibiotics is crucial for managing the morbidity and mortality of the infection (Ferrer et al., 2014; Falcone et al., 2020). According to the report of the Chinese National Bacterial Drug Resistance Monitoring Network, *Pseudomonas aeruginosa* isolated from blood samples during 2014–2019 had the highest composition proportion, the second was *A.baumannii* (Wu et al., 2021). In our previous studies, *K.pneumonia* was the most commonly isolated Gram-negative bacteria in affected intensive care unit (ICU) patients, the second was *E. coli*, and *A.baumannii* was in the third place (Wu et al., 2022). *A. Baumannii* is an important cause of bacteremia in hospitals, and is associated with high mortality, ranging from 17 to 63% (Cisneros and Rodríguez-Baño, 2002). It is a non-fermentation, motion-negative, oxidase-negative Gram-negative bacillus, which often causes multiple site infections in the blood, lower respiratory tract, urinary tract, and wound (Munoz-Price and Weinstein, 2008).

The increasing number of MDR and even pan drug resistant (PDR) strains has brought great difficulties to clinical anti-infection treatment. In recent 2 years, the drug resistance rate of *A. baumannii* has increased significantly, and the PDR *A. baumannii* is almost incurable (Munoz-Price and Weinstein, 2008), which has become a focus of medical attention. Therefore, bacterial drug resistance monitoring has positive significance for understanding the changes of drug resistance and guiding clinical rational drug use. Moreover, serious risk factors for poor prognosis of *A. Baumannii* bacteremia include basic diseases, bacteremia source for pneumonia, septic shock and disseminated intravascular coagulation, surgery, invasive operation, mechanical ventilation, decreased immunity, length of stay in the ICU, length of hospital stay, and the extension of the total length of hospital stay, etc. (Vázquez-López et al., 2020). Although these prognostic factors play an important role in predicting clinical outcomes, objective quantitative assessments of severity are more important in treatment decisions. Several organ dysfunction scoring systems have been developed to assess the prognosis of critically ill patients (Marshall et al., 1995; Le Gall et al., 1996; Vincent et al., 1996). The Acute Physiology and Chronic Health Evaluation II (APACHE II) and Sequential Organ Failure Assessment (SOFA) are the two most commonly used scoring systems for organ dysfunction. Although these studies were originally intended to classify and quantify the extent of organ failure rather than predict patient outcomes, several studies have demonstrated a clear relationship between organ dysfunction and mortality (Ferreira et al., 2001; Kajdacsy-Balla Amaral et al., 2005). Although an updated version of the APACHE II prediction model has been proposed (Zimmerman et al., 2006), and studies by Varghese YE et al have confirmed that APACHE IV scores are superior to APACHE II scores (Varghese et al., 2017), many intensive care units (ICUS) still use the APACHE II scores introduced in 1985. The SOFA Score describes the extent of organ dysfunction over time and estimates the incidence of sepsis in ICU patients (Vincent et al., 1998). The SOFA scoring system assigns a score of 1–4 points to the respiratory, circulatory, renal, hematology, hepatic, and central nervous systems, depending on the extent of dysfunction.

Previous studies have shown that APACHE II score and SOFA score are associated with the occurrence and prognosis of *A.baumannii* bacteremia (Lee et al., 2022). But comparisons of the predictive effects of these two indicators are relatively few. This study retrospectively analyzed the risk factors, clinical characteristics, bacterial resistance and prognostic factors of 110 cases of *A. Baumannii* bacteremia in our hospital, compared the survival time between MDR and Non-MDR patients, and validated the effectiveness of the APACHE II and SOFA scoring systems in predicting the outcome of patients with *A. baumannii* bacteremia based on assessment scores taken at the onset of bacteremia.

## Materials and methods

### General data

This retrospective study was conducted in Tianjin Medical University General Hospitalin Tianjin, China, from February 2017 to March 2022 and was approved by the hospital’s ethics committee (NO. IRB2022-WZ-077). This tertiary teaching hospital is a 2,468-bed facility located in Tianjin. A total of 110 patients were diagnosed with *A. Baumannii* bacteremia were collected. All patients over 18 years of age with a confirmed *A. Baumannii* bacteremia during hospitalization was included in this study. Blood culture is the golden criterion for the diagnosis of BSI and was performed for patients with infectious symptoms, such as fever, cold, shivering, and low blood pressure, before administering antimicrobial therapy. The specimens were sent to the microbiology laboratory, and the VITEK-2 compact automated system was used for bacterial identification and antibiotic susceptibility testing. The antimicrobial susceptibility tests were interpreted according to the guidelines of the Clinical and Laboratory Standards Institute (CLSI) (Dalyan Cilo and Ener, 2021). For results not included in CLSI, the guidelines prescribed by the European Committee on Antimicrobial Susceptibility Testing (EUCAST) were referred (Giske et al., 2022). Our hospital has developed a critical value reporting system, according to which, if the blood culture results are positive, the microbiology laboratory must report Gram-positive, Gram-negative, or candida as soon as possible; the rapid reporting could direct the clinician’s decision and reduce the delay in initiating the antibiotic treatment. However, generally, it takes 3–5 days to get the final results.

The patients’ data included: (1) sex, age, department of admission, Glasgow coma score (GCS), treatment and outcome were extracted from health information system (HIS). Basic diseases: hypertension, diabetes, renal insufficiency, malignant tumor, etc. Invasive operations: invasive mechanical ventilation [tracheal intubation or tracheotomy], deep vein catheterization, CRRT (continuous renal replacement therapy), surgery, bronchofibroscope, etc. (2) Laboratory information system (LIS) provides the results of microbial isolation, antimicrobial resistance, blood routine, serum creatinine and arterial blood gas analysis of patient specimens. The highest Acute Physiology and Chronic Health Evaluation II score (APACHE II), SOFA score were calculated within 48 h after blood culture. The following conditions were excluded: (1) Those who were transferred out within 48 h after admission, or died or gave up treatment due to various factors; (2) Incomplete clinical data; (3) Persons under the age of 18. The results of drug sensitivity were checked by Lis (Laboratory Information System). They were divided into survival group and non-survival group according to disease outcome.

### Diagnostic criteria

Blood culture and identification of *A. baumannii* strains shall be conducted in strict accordance with national Clinical Examination Procedures. The diagnostic criteria for bacteremia refer to the Diagnostic Criteria for Nosocomial Infection issued by the Ministry of Health in 2001. Bacteremia occurring 48 h after admission was defined as nosocomial bacteremia. Continuous culture of the same pathogen within 7 days after the occurrence of bacteremia in the same patient was regarded as one case of infection.

### Definition of drug resistance of *Acinetobacter baumannii*

Carbapenem-resistant *A.baumannii* (CRAB) refers to antimicrobial resistance to imipenem and meropenem at the same time. Carbapenem-susceptible *A.baumannii* (CSAB) refers to *A.baumannii* which is susceptible to both imipenem and meropenem. Multidrug-resistant (MDR) acinetobacter refers to the drug resistance of three or more types of antibacterial drugs (mainly cephalosporins and carbapenems against *Pseudomonas*, compound preparations containing β lactamase inhibitors, fluoroquinolones, and aminoglycosides) that have potential antibacterial activity against the bacterium. Extensively drug-resistant (XDR) acinetobacter refers to strains that are sensitive only to tigecycline and/or polymyxins. PDR acinetobacter refers to the strain that is resistant to all available antibacterial drugs in China (Falagas et al., 2006; Magiorakos et al., 2012).

### Statistical analysis

SPSS 26.0 statistical software was used for data processing. The measurement data with normal distribution and homogeneity of variance was expressed as mean ± standard deviation (−χ ± S), and two independent samples *t*-test was used. The measurement data with non-normal distribution and uneven variance were represented by median (quartile) [M (QL, QU)], and rank-sum test was used for comparison between groups. The statistical and categorical variables were expressed by case number (constituent ratio), and χ^2^-test was used between groups. Univariate analysis was used to analyze the risk factors affecting death of patients with bacteremia, and factors with *P* < 0.05 were included in multivariate logistic regression analysis to screen out independent predictors of death of patients with bacteremia. Statistical software of R Studio language was used to analyze the correlation and decision tree of the collected data to determine which factor was dominant in the prognosis of patients with *A.baumannii* bacteremia. Kaplan-Meier method was used for survival analysis, and Log-rank test was used to compare the differences between MDR and non-MDR groups. The receiver operator characteristic curve (ROC curve) of APACH II score and SOFA score was plotted to find the optimal cut-off point and determine the threshold for predicting death. For each ROC analysis, the cut-off values, sensitivity, specificity were calculated. The best cut-off point was determined when the point yielded the best specificity and sensitivity. The Youden index (sensitivity + specificity –1) was calculated, and the maximum value was used to identify the optimal cut-off. MedCalc software was used for ROC analysis. The area under ROC curve (AUC) was compared using Hanley and McNeil *Z*-test. The correlation of APCHE II and SOFA was assessed by linear regression with Pearson analysis. All *p*-values were 2-tailed, and *p* < 0.05 was considered to be statistically significant.

## Results

### General condition of patients

**FIGURE 1:**
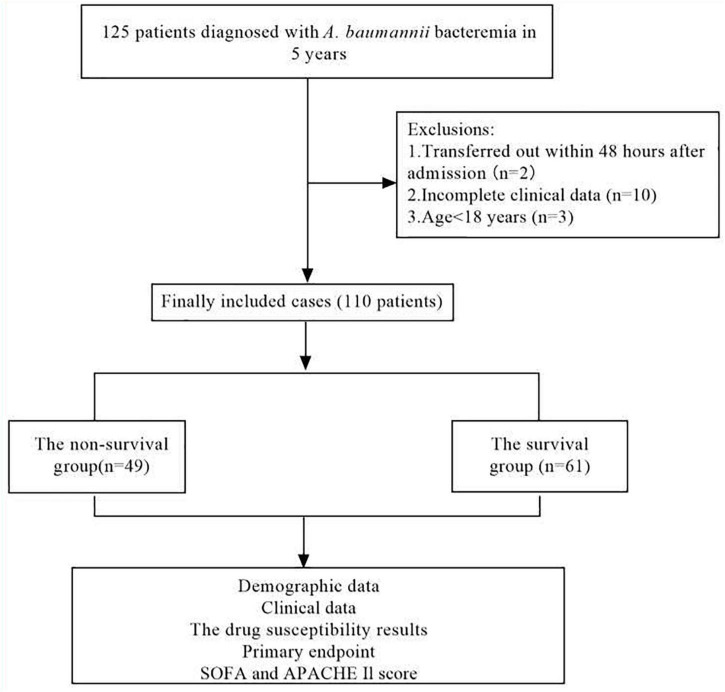
Flow chart for patient selection. A total of 125 *A.baumannii* blood culture positive specimens were analyzed for drug resistance and the clinical data. After excluding 15 patients with incomplete clinical data, 110 cases of *A.baumannii* bacteremia remainder were included in the clinical data analysis.

**FIGURE 2:**
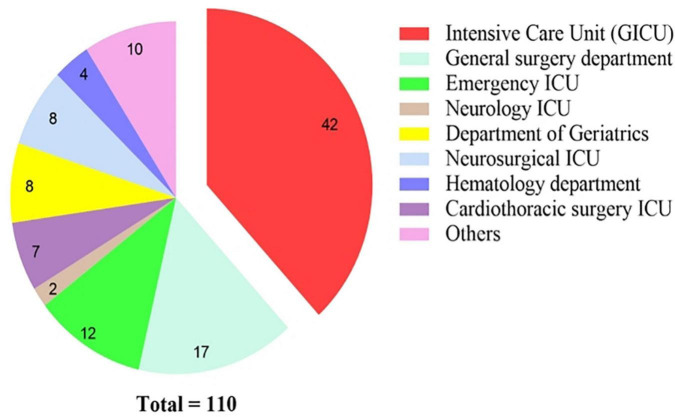
Distribution and constitution of departments for *A. baumannii* bacteremia.

**FIGURE 3:**
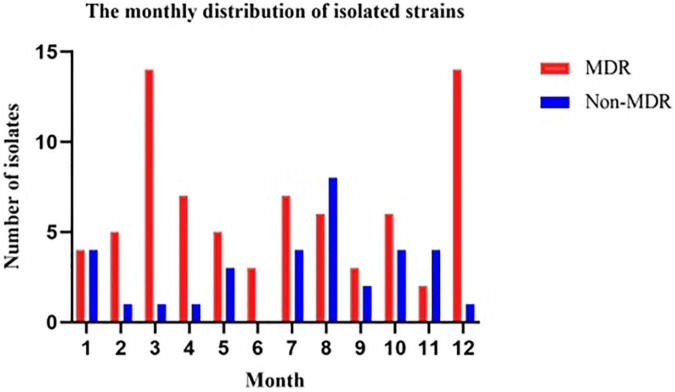
Number of *A. baumannii* isolates recovered from patients from February, 2017 through March 2022 by month. The isolated month distribution of MDRAB differed significantly from non-MDRAB. For MDRAB isolates, the most common months were March and December, whereas for non-MDRAB isolates, the most common month was August. MDRAB, multidrug-resistant *A. baumannii.*

From February 2017 to March 2022, a total of 110 patients were diagnosed with *A. baumannii* bacteremia with hospitalization duration > 48 h in various departments of our hospital (Figure 1), among which 74 were male, accounting for 67.3%; female 26 cases (32.7%). The age ranged from 28 to 94 years, with an average of (63.5 ± 15.1) years. Patients were distributed in 17 different departments (Figure 2), among them, 42 cases (38.2%) were in intensive care unit (General ICU), followed by 17 cases (15.5%) in general surgery department and 12 cases (10.9%) in Emergency ICU. The *A. baumannii* strains were mostly isolated in March and December, whereas Non-MDRAB strains were isolated in August (Figure 3).

Basic diseases mainly include: hypertension, coronary heart disease, diabetes, renal insufficiency, malignant tumor and so on. Among them, 46 cases (41.81%) had 3 basic diseases or more. There were 21 cases (19.09%) with two basic diseases, 24 cases (21.82%) had one basic disease. An average of (29.13 ± 5.54) days after hospitalization, the blood culture was *A. baumannii*.

### Drug sensitivity results of *Acinetobacter baumannii* bacteremia

Among 110 patients with *A. baumannii* bacteremia, 3 cases (2.7%) were PDR, 76 cases (69.1%) were MDR, and 77 cases (70.0%) were CRAB. Among them, the drug resistance rate of amtronam was 100.0%. The drug resistance rates of ticacillin/clavulanic acid, piperacillin/tazobactam, ceftazidime, and cefepime were 67.0, 69.1, 67.0, and 60%, respectively. The drug resistance rates to imipenem, meropenem, levofloxacin, amikacin, and cefoperazone/sulbactam were 70.0, 67.0, 55.5, 20.0, and 41.0%, respectively. The drug resistance rates to tigecycline and colistin were 3.6 and 1.1%, respectively (Table 1).

**TABLE 1:** *In vitro* susceptibility results of 110 patients with *A. baumannii* bloodstream infection.

Antibiotic types	Cases	R [cases (%)]	I [cases (%)]	S [cases (%)]
Levofloxacin	110	61 (55.5)	13 (11.8)	36 (32.7)
Ceftazidime	88	59 (67.0)	1 (1.1)	28 (31.8)
Imipenem	110	77 (70.0)	0 (0)	33 (30.0)
Piperacillin/tazobactam	110	76 (69.1)	2 (1.8)	32 (29.1)
Ciprofloxacin	110	75 (68.2)	0 (0)	35 (31.8)
Minocycline	88	13 (14.8)	33 (37.5)	42 (47.7)
Tobramycin	110	37 (33.6)	1 (0.9)	72 (65.5)
Trimethoprim/sulfamethoxazole	110	55 (50.0)	0 (0)	55 (50.0)
Meropenem	88	59 (67.0)	0 (0)	29 (33.0)
Aztreonam	110	110 (100)	0 (0)	0 (0)
Cefepime	110	66 (60.0)	10 (9.1)	34 (30.9)
Ticarcillin/clavulanic acid	88	59 (67.0)	0 (0)	29 (33.0)
Cefopetazone/sulbactam	100	41 (41.0)	10 (10.0)	49 (49.0)
Doxycycline	88	56 (63.6)	0 (0)	32 (36.4)
Amikacin	110	22 (20.0)	2 (1.8)	86 (78.2)
Tigecycline	110	4 (3.6)	21 (19.1)	85 (77.3)
Colistin	88	1 (1.1)	0 (0)	87 (98.9)

### Analysis of risk factors affecting the prognosis of 28 days of patients with *Acinetobacter baumannii* bacteremia

**FIGURE 4:**
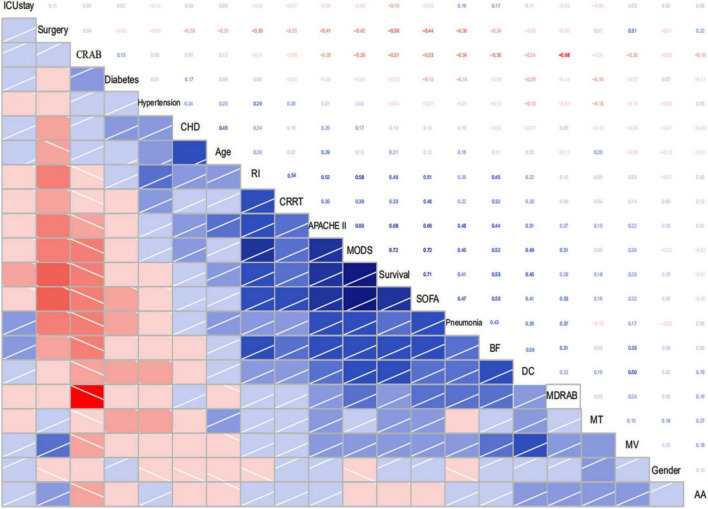
Correlogram of my data intercorrelations. CRAB, Carbapenem resistant *A. baumannii*; CHD, Coronary heart disease; RI, Renal insufficiency; BF, Bronchofibroscope; DC, Deepvein catheterization; MDRAB, Multi-drug resistant *A. baumannii;* MT, Malignant tumor; MV, Mechanical ventilation; AA, Acute abdomen.

In our study, a total of 110 patients with positive *A. baumannii* blood culture were included, and divided into two groups according to clinical outcomes at 28 days, 61 patients in the survival group and 49 patients in the non-survival group. There were no significant differences in sex, proportion of basic diseases such as hypertension, diabetes and cardiovascular disease between survival group and non-survival group (all *P* > 0.05). The age of patients in non-survival group (67.06 ± 13.28 years) was higher than that in survival group (60.69 ± 16.09 years), *P* < 0.05. Univariate analysis of prognosis indicated that the risk factors affecting the prognosis of 28 days of patients with *A. baumannii* bacteremia included: Age of patients, ICU stay, surgery, mechanical ventilation, CRAB and MDR *A. baumannii* bacteremia indicated by blood culture, MODS, APACHE II score, SOFA score and so on (Table 2). Multivariate logistic regression analysis showed that, APACHE II score and SOFA score were independent risk factors for 28d prognosis of *A. baumannii* bacteremia, while surgery was a protective factor for *A. baumannii* bacteremia (Table 3). Statistical software of R Studio analyzed the correlation of the collected data, the results showed that APACHE II (*r* = 0.68) and SOFA (*r* = 0.72) were highly correlated with the prognosis of patients (Figure 4).

**TABLE 2:** Univariate analysis of the risk factors predicting 28-day death in patients with *A. baumannii* bacteremia.

	Survival (*n* = 61)	Non-survival (*n* = 49)	χ2/t/z value	*P*-value
Age (mean ± SD)/years	60.69 ± 16.09	67.06 ± 13.28	–2.175a	0.030
Male [*n* (%)]	43 (70.49%)	31 (63.27%)	0.645b	0.422
ICU stay [*n* (%)]	42 (68.85%)	45 (91.84%)	8.68b	0.003
Hospital stay [d, M (Ql,Qu)]	40.0 (20.0, 66.0)	29.0 (12.0, 43.5)	–2.271c	0.023
Pneumonia [*n* (%)]	37 (60.66%)	47 (95.92%)	18.720b	0.000
**No. of recent invasive procedures (%)**				
Surgery [*n* (%)]	43 (70.49%)	10 (20.41%)	27.300b	0.000
Mechanical ventilation [*n* (%)]	40 (65.57%)	42 (85.71%)	5.809b	0.016
Deep vein catheterization [*n* (%)]	34 (55.74%)	47 (95.92%)	22.598b	0.000
Bronchofibroscope [*n* (%)]	16 (26.67%)	39 (81.25%)	31.791b	0.000
MDRAB [*n* (%)]	35 (57.38%)	41 (83.67%)	15.205b	0.002
CRAB [*n* (%)]	35 (57.38%)	42 (85.71%)	10.390b	0.001
**Underlying diseases**				
Hypertension [*n* (%)]	33 (55.00)	25 (51.02)	0.172b	0.679
Diabetes [*n* (%)]	15 (24.59%)	11 (22.45%)	0.069b	0.793
Coronary heart disease [*n* (%)]	19 (31.15)	20 (40.82)	1.110b	0.292
Renal insufficiency [*n* (%)]	11 (18.03)	31 (63.27%)	23.554b	0.000
Malignant tumor [*n* (%)]	18 (29.51%)	23 (46.94%)	3.531b	0.060
CRRT [*n* (%)]	5 (8.20%)	17 (34.69%)	11.924b	0.001
MODS [*n* (%)]	5 (8.20%)	39 (79.59%)	57.711b	0.000
SOFA score [M (Ql, Qu)]	5.00 (3.00, 6.00)	10.00 (8.00, 12.5)	7.391c	0.000
APACHE II score [M (Ql, Qu)]	14.00 (10.00, 19.00)	30.00 (23.50, 33.00)	7.099c	0.000

ICU, Intensive Care Unit; SOFA, Sequential Organ Failure Assessment score; APACHE II, Acute Physiology and Chronic Health Evaluation II score; MODS, Multiple Organ Dysfunction Syndrome; CRAB, Carbapenem-resistant *A.baumannii*; MDR, Multidrug-resistant *A.baumannii*; CRRT, Continuous Renal Replacement Therapy; t, Student *t*-test; χ2, Chi-square test; z, Rank-sum test; *p*-value, < 0.05 was considered as statistically significant; a for t, b for χ^2^; c for z.

**TABLE 3:** Multivariate logistic analysis of the risk factors predicting 28-day death in patients with *A. baumannii* bacteremia.

Variable	β	S.E.	Wald χ^2^	OR value	95%CI	*P*-value
SOFA	0.357	0.177	4.07	1.429	1.01 ∼ 2.022	0.044
APACHE II	0.116	0.052	5.047	1.123	1.015 ∼ 1.242	0.025
Surgery	–2.599	1.066	5.946	0.074	0.009 ∼ 0.6	0.015

SOFA, Sequential Organ Failure Assessment score; APACHE II, Acute Physiology and Chronic Health Evaluation II score.

### Comparison of survival between multidrug-resistant and non-multidrug-resistant *Acinetobacter baumannii* bacteremia patients

**FIGURE 5:**
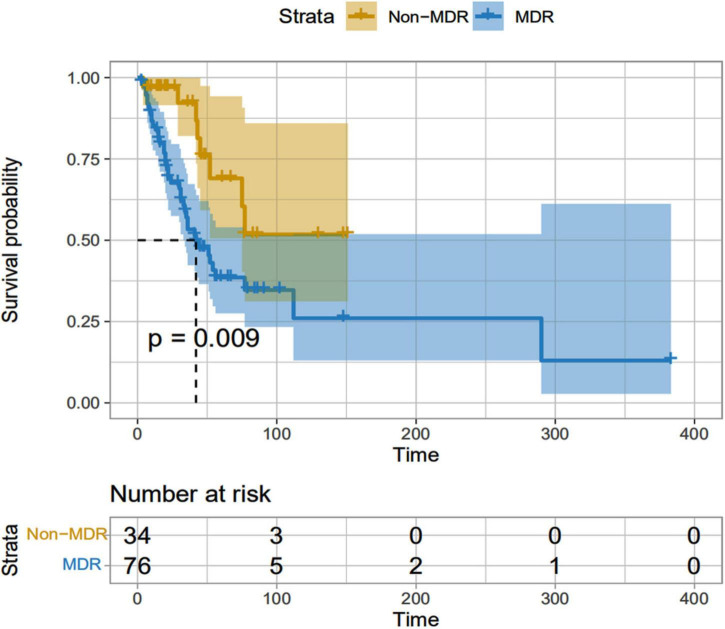
MDR and Non-MDR survival curves of patients infected with A. baumannii bacteremia. MDR, Multidrug-resistant.

All patients enrolled had nosocomial infection, and the average length of hospital stay before infection was 3∼407 (29.13 ± 58.06) days. The non-survival group was (32.08 ± 62.36) days, and the survival group was (26.75 ± 54.78) days. There were 69 cases (79.3%) of MDR in ICU patients and only 7 cases (30.4%) of patients who were not admitted to ICU. The Kaplan-meier method was used for survival analysis. There were 76 cases in MDR group, 41 cases died, and the survival curve decreased sharply. There were 34 cases in the Non-MDR group, and 8 cases died. The decline of the survival curve was relatively gentle, and the survival time of the MDR group was shorter than that of the Non-MDR group (mean survival time 43.87 d vs. 47.06 d, *P* = 0.009) ((Table 4 and Figure 5).

**TABLE 4:** Comparison of mortality and survival between MDR and Non-MDR *A. baumannii* bacteremia patients.

Group	Death cases [*n* (%)]	Mean survival time (*d*)	95% CI	Log-rank χ^2^-value	*P*-value
MDR (*n* = 76)	41 (53.95)	43.87	66.922∼166.435	6.815	0.009
Non-MDR (*n* = 34)	8 (23.53)	47.06	78.446∼128.473		

MDR, Multidrug-resistant.

### Comparison of the prognostic value of area under receiver operator characteristic curve between sequential organ failure assessment score and acute physiology and chronic health evaluation II score

**FIGURE 6:**
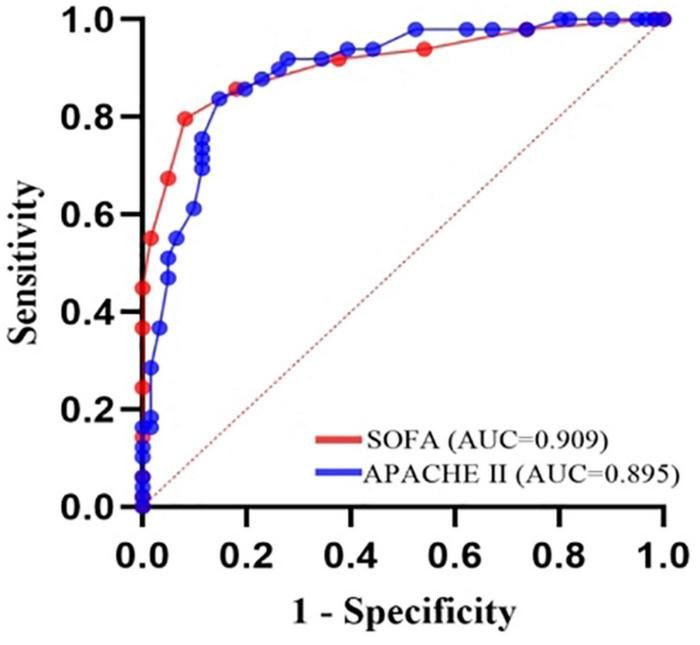
Receiver-operating characteristic (ROC) plot of 28-day mortality predictions using the organ failure systems for 110 *A. baumannii* bacteremia patients. SOFA score, AUC = 0.909, cutoff point 7, sensitivity 79.6%, specificity 91.8%. APACHE II score, AUC = 0.895, cutoff point 21, sensitivity 83.7%, specificity 85.2%.

**FIGURE 7:**
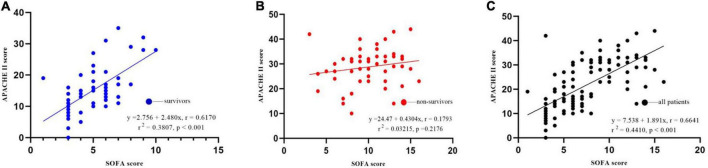
Correlation between SOFA and APACHE II scores for 28-day mortality. **(A)** Survival group; **(B)** non-survival group; **(C)** all patients.

Prediction of death from *A. baumannii* bacteremia by SOFA score and APACHE II score according to ROC curve, the critical value of SOFA score for predicting death from *A. baumannii* bacteremia at 28d was 7 points, and the area under ROC curve was 0.909, 95% CI (0.839–0.955), *P* < 0.001. The sensitivity and specificity were 79.6 and 91.8%, respectively. The critical value of APACHE II score for predicting 28d death of patients with *A. baumannii* bacteremia was 21 points, the area under ROC curve was 0.895, 95% CI (0.822–0.945), *P* = 0.000, the sensitivity was 83.7%, and the specificity was 85.2% [Table 5 and Figure 6]. In conclusion, SOFA score and APACHE II score have high diagnostic value for 28d prognosis of *A. baumannii* bacteremia patients. The AUC difference between the two was 0.0144, *Z*-value was 0.409, *P* = 0.6828, and there was no statistically significant difference between SOFA score and APACHE II score (Table 6). In addition, there was a significant correlation between SOFA and APACHE II scores (*p* < 0.001) in the survival group and all patients (Figure 7). However, there was an exception in non-survivors with in 28d, which may be attributed to the small number of cases (*n* = 41).

**TABLE 5:** Discriminatory abilities of the APACHE II and SOFA scores in predicting death of the *A. baumannii* bacteremia patients.

Variable	AUC	SE^a^	95% CI^b^	Sensitivity (%)	Specificity (%)	Cut-off scores
APACHE II	0.895	0.0307	0.822–0.945	83.7%	85.2%	21
SOFA	0.909	0.0298	0.839–0.955	79.6%	91.8%	7

^a^Hanley and McNeil (1982); ^b^Binomial exact.

**TABLE 6:** Pairwise comparison of ROC curves.

APACHE II ∼ SOFA	
Difference between areas	0.0144
Standard error^a^	0.0352
95% confidence interval	–0.0546 to 0.0834
z statistic	0.409
Significance level	*P* = 0.6828

^a^Hanley and McNeil (1983).

### The decision tree model was used to predict the prognosis of *Acinetobacter baumannii* bacteremia

**FIGURE 8:**
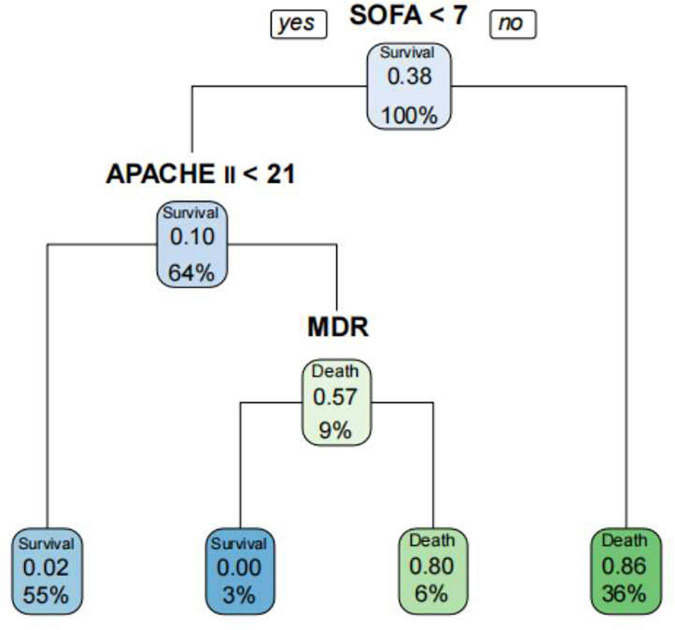
Prediction of the prognosis of patients with *A. baumannii* bacteremia by decision tree model. The decision tree had four depth and four leaf nodes, SOFA, APACHE II, and MDR were included as discriminating factors in the decision tree for predicting patients with *A. baumannii* bacteremia.

The decision tree model is a tree structure composed of root node, branch node and leaf node, which reflects the mapping relationship between features and tags (Che et al., 2011). In the decision tree algorithm, in order to avoid model overfitting, cp value (cp = 0.035714, branch = 1, type = 1) was adopted in this study for pruning. The decision tree model was used to predict the outcome of *A. baumannii* bacteremia. The decision tree had four depth and four leaf nodes, APACHE II, SOFA, and MDR were included as discriminating factors in the decision tree for predicting the outcome of *A. baumannii* bacteremia. We fitted APACHE II, SOFA, and MDR by decision tree (Figure 8), and the ability to identify accurate accuracy of the prognosis of *A. baumannii* bacteremia was 0.879. The model confirmed that SOFA and APACHE II play an important role in the prognosis assessment of patient with *A. baumannii* bacteremia.

## Discussion

In this study, 110 patients with *A. baumannii* bacteremia were all nosocomial infections, and most of them originated from ICU. It is worth noting that the population in this study was relatively old (average age was 68.9 years old), mostly male (67.3%), and most of the patients in the non-survival group were elderly patients complicated with pulmonary infection and MODS. At the same time, most of the bloodstream infections of *A. baumannii* bacteremia in this study were CRAB and MDRAB, 3 cases (2.7%) were PDR, the drug resistance rates to imipenem and meropenem were 70.0 and 67.0%, respectively. The survival time of patients in MDR group was significantly shorter than that in Non-MDR group. The survival group and the non-survival group also showed statistically significant differences in airway opening, invasive mechanical ventilation, deep venous catheterization and other invasive procedures. Invasive treatment and examination are one of the risk factors for bloodstream infection of *A. baumannii* bacteremia. This study showed that APACHE II score and SOFA score of patients in the survival group were significantly lower than those in the non-survival group. Analysis of the optimal sensitivity and specificity of ROC curve showed that patients with APACHE II score > 21 and SOFA score > 7 had an increased risk of death.

At present, the incidence of bacteremia is already high worldwide, and the incidence, mortality and medical costs of bacteremia are still increasing (Kern and Rieg, 2020). Blood culture is still the “gold standard” for the diagnosis of bacteremia (Adrie et al., 2017), but it always has a low positive rate and a long time. Once pathogens or toxins enter the blood in large numbers and cause systemic infection, the fatality rate of patients will increase significantly (Tang et al., 2018). Suspicion of bacteremia warrants immediate blood tests, and if bacteremia is confirmed, pathogen-based treatment should be initiated immediately. Bacteremia due to *A. baumannii* is characteristically a nosocomial infection with a fatality rate of up to 37%. Bacteremia, followed by respiratory tract and surgical wound infections, is the most significant infection caused by *A. baumannii* (Chiu et al., 2015). A total of 110 patients with *A. baumannii* bacteremia were included in this study, including 61 patients in the survival group and 49 patients in the non-survival group. The case fatality rate on 28 days was 44.55%, which was similar to the results reported by Huang et al. (2020) and Yang et al. (2021). In this study, we found that the detection of *A. baumannii* was mainly concentrated in the ICU. A total of 87 patients (79.1%) were admitted to the ICU, including 42 patients (68.9%) in the survival group and 45 patients (91.84%) in the non-survival group were admitted to ICU. Therefore, there is a significant increase in mortality among patients admitted to the ICU and with *A.baumannii* bacteremia, and the causes for this are analyzed as follows (Marchaim et al., 2017; Tanguy et al., 2017; Lopez-Gigosos et al., 2019): (1) ICU patients are often complicated with MODS, complicated disease, low body resistance, long-term broad-spectrum antibiotic exposure, high risk of composite end point adverse events and poor prognosis; (2) ICU patients need to take a variety of invasive diagnosis and treatment measures, the immune barrier is destroyed, increase the risk of pathogenic bacteria into the blood; (3) The majority of patients with tracheal intubation/tracheotomy in ICU have weak cough reflex and decreased airway secretion clearance ability, resulting in the growth and reproduction of a large number of bacteria and the production of toxins; (4) CRAB has strong adhesion ability and is easy to colonize various medical equipment in ICU. It can cause cross transmission and even outbreak through medical operations of medical staff. According to the survey, the average fatality rate of ICU in tertiary hospitals in China was only 12.80% (Pei et al., 2016), which indirectly indicated that *A. baumannii* bacteremia significantly increased the fatality rate of ICU. The ICU of our hospital is a General ICU, with the highest incidence of *A.baumannii* bacteremia, because the patients admitted to the GICU include medical severe, surgical severe, acute trauma, major surgery, and patients with critical condition, low immune function, and long hospital stay. *A. baumannii* bacteremia has become an important problem of ICU nosocomial infection, which should be paid more attention to by clinicians due to its difficulty in treatment and high mortality. Therefore, we believe that comprehensive measures should be taken to address the above risk factors, including: the effective strategies to prevent and control *A. baumannii* infection are environmental isolation, strict disinfection of bed units, strengthening hand hygiene education and execution supervision, minimizing mechanical ventilation time, reducing ICU stay time, strengthening the drainage of sputum, secretions and other body fluids, and rational use of antibiotics.

In our study, patients in the non-survival group were older than those in the survival group, therefore, advanced age is one of the risk factors for death of *A. baumannii* bacteremia, which is related to the increase of basic diseases, decreased immunity and weakened resistance with the increase of age. In addition, male patients have a higher mortality rate, and patients with lung infection have a higher mortality rate. Male patients may smoke more, and smoking will cause damage to respiratory epithelial cells and damage the respiratory mucosal barrier. When immunity is low, *A. baumannii* fixed in the respiratory tract can cause respiratory tract infection or even lung infection. Patients with pulmonary infection are more likely to receive broad-spectrum antibiotics and other invasive procedures, and these factors interact with each other to result in longer hospital stays, poor prognosis, and high mortality. Clinicians should comprehensively consider the patient’s condition and choose reasonable drug treatment to improve the curative effect. Previous studies have shown that the mortality of drug-resistant *A. baumannii* bloodstream infection is as high as 58.24–79.8% (Kim et al., 2012; Lee et al., 2014; Liu et al., 2016). It was reported that the high drug resistance rate of *A. baumannii* was related to its complex drug resistance mechanism (Nordmann and Poirel, 2002), and the extensive use of broad spectrum antibacterial agents and experiential drug use could also induce the increase of drug resistance of *A. baumannii*. In this study, to further analyze the impact of bacterial resistance on the prognosis of patients, patients with *A.baumannii* bloodstream infection were divided into MDR group (76 cases) and Non-MDR group (34 cases). Kaplan-Meier survival curve showed that the prognosis of patients in MDR group was significantly worse than that in Non-MDR group (*P* < 0.05). In addition, in this study, the drug resistance rates of the isolated MDR-AB strains to the second and third generation cephalosporins, carbapenems, fluoroquinolones and aminoglycosides were all over 80%, and they were only sensitive to tigecycline and colistin. Therefore, the antimicrobial agents available for clinical treatment of MDR-AB infection are very limited. Carbapenems have been considered as the “antibiotics of last resort” in the treatment of Gram-negative bacterial infections, and CRAB has been widely prevalent around the world and has been listed by WHO as one of the drug-resistant bacteria posing the greatest threat to modern medicine (Shrivastava et al., 2018). In 2019, CHINET results showed that the drug resistance rate of *A. baumannii* to imipenem and meropenem increased to 77.7 and 79.0%, and even the drug resistance rate of *A. baumannii* to carbapenems was as high as 80.1 ∼ 80.5% in some regions, the drug resistance rate was higher than that in this study. This may be related to the environmental health monitoring, nosocomial infection prevention and control policies adopted by our hospital. At the same time, the drug sensitivity results of this study showed that tigecycline and colistin remained highly sensitive to *A. baumannii*. Although tigecycline is highly sensitive to MDRAB, the peak serum concentration of tigecycline at conventional dose is low (Cooper et al., 2011), and the protein binding rate is high (80%), so the concentration of free blood drug is low, and it is not recommended for the treatment of bloodstream infection (Bi et al., 2017). Increasing the dose can increase the blood concentration. Previous studies have suggested that doubling the dose can improve the treatment effect of nosocomial acquired pneumonia and other infections (Falagas et al., 2014; Ni et al., 2016), but the treatment effect of MDRAB bloodstream infection needs to be further explored. Katip et al. (2021) collected 383 CRAB patients over 6 years to evaluate 30-day survival and nephrotoxicity in critically ill patients treated with non-loading vs. loading doses of Colistin methanesulfonate in the treatment of CRAB infection. At the 30th day of treatment, the survival rate of patients in the loading doses Colistin methanesulfonate group was 1.70 times that of patients in the non-loading doses group. Clinical response was significantly higher in the loading doses Colistin methanesulfonate group than non-loading doses Colistin methanesulfonate group. In addition, a microbiological response-eradication of pre-treatment isolated pathogens in post-treatment cultures in patients with loading doses of colistin methanesulfonate was 1.57 times that of patients with non-loading doses Colistin methanesulfonate. Additionally, there was a significant difference in nephrotoxicity between loading doses of colistin methanesulfonate and non-loading doses Colistin methanesulfonate (aHR, 1.57, 95% CI, 1.14–2.17, *p* = 0.006). Based on these results, loading doses of colistin methanesulfonate should be used to increase the opportunity for patients to achieve favorable outcomes. However, loading doses of colistin methanesulfonate was found associated with an increase in nephrotoxicity, so renal function should be closely monitored when loading doses of colistin methanesulfonate was administered. Although the application of colistin and tigecycline in clinical studies is still controversial, colistin and tigecycline almost always showed superiority over other antibiotics in the fight against MDRAB. In clinical use, these two antibiotics remain the last defense against these stubborn bacteria. Gram-negative bacteria, including *A.baumannii*, are increasingly recognized as exhibiting seasonal trends in bloodstream infection incidence; that is, significantly higher rates of *A. baumannii* bacteremia were observed during the summer months (Perencevich et al., 2008; Eber et al., 2011; Richet, 2012). However, in our study, the *A. baumannii* strains were mostly isolated in March and December, whereas non-MDRAB strains were isolated in late summer months (August) (Figure 3). These results can influence clinical diagnosis and empiric antibiotic treatment, and can be used as an important reference for antibiotic selection in patients with *A. baumannii* infection.

This study retrospectively compared the predictive ability of APACHE II and SOFA scoring systems in the prognosis assessment of patients with *A. baumannii* bacteremia. In the sepsis 3.0 definition, the SOFA score is recommended as a clinical diagnostic criterion for sepsis and can be used to evaluate the prognosis of patients with ICU infection (AUC 0.740) (Singer et al., 2016). In addition, the SOFA score can evaluate the severity of organ dysfunction in patients with sepsis. Studies have shown that SOFA score has a good predictive ability for the prognosis of patients with sepsis and septic shock in the emergency department (Jones et al., 2009), severe sepsis in the ICU (Cui et al., 2014), obstetric ICU and cardiothoracic surgery. SOFA score also has a high application value in other critically ill patients. Adam et al. (2013) screened retrospective charts of 43 patients hospitalized in the intensive care unit with severe acute pancreatitis. Acute Physiology and Chronic Health Evaluation II, SOFA and modified Ranson’s scores were calculated on admission, and SOFA score was recorded at weekly intervals during the intensive care unit stay. They found that continuous organ failure assessment scores were significantly associated with mortality. All patients with a score ≥ 11 on any chronological organ failure assessment in the intensive care unit had a higher risk of mortality (80% sensitivity, 79% specificity, ROC = 0.837). And no statistically significant associations were found between Acute Physiology and Chronic Health Evaluation II scores and mortality. Vincent et al. (1996) conducted the first retrospective analysis of SOFA score in the first 24 h of 1,643 infected patients, and the results showed that SOFA score was well correlated with mortality. Although SOFA alone has been considered a predictor of mortality in previous studies, the limitation of SOFA is that it does not take into account the patient’s age and previous medical history, so there are limitations in the assessment (Yang et al., 2021). For critically ill patients, APACHE II score is recommended to predict the severity of the disease, and the higher the APACHE II score, the more severe the disease (Rhodes et al., 2017). There are many domestic and foreign reports that APACHE II score can effectively evaluate the prognosis and length of ICU stay of ICU patients (Naved et al., 2011). The APACHE II score has also been used to assess the prognosis of cardiovascular, respiratory and nervous system diseases (Li et al., 2014), and its predictive power has also been recognized. Yang et al. (2021) investigated the prognostic value of platelet count (PLT), four coagulation items, APACHE II score and SOFA score in patients with bloodstream infection. They found that PLT and coagulation are helpful to evaluate the prognosis of ICU patients with blood flow infection. APACHE II score and SOFA score are directly related to the prognosis of patients with blood flow infection. This study showed that SOFA score and APACHE II score in the non-survival group were significantly higher than those in the survival group, suggesting that the severity of disease in the non-survival group was significantly different from that in the survival group. Multivariate Logistic regression analysis also showed that SOFA score and APACHE II score were independent risk factors for 28-day death in patients with bacteremia. ROC curve analysis confirmed that SOFA score (AUC 0.909) and APACHE II score (AUC 0.895) were reliable factors for predicting 28-day death in patients with bacteremia. Moreover, the two scores were highly correlated and equally effective at predicting mortality. We also found that SOFA > 7 and APACHE II > 21 were associated with higher 28-day mortality. Therefore, if the SOFA score was ≤ 7 or APACHE II ≤ 21 at the onset of bacteremia, patients were likely to survive for more than 28 days. The AUC difference was 0.0140, *Z*-value was 0.3245, *P* = 0.7455, and there was no statistically significant difference between SOFA score and APACHE II score. Chen et al. (2011) compared the effectiveness of SOFA score and APACHE II score in predicting the prognosis of *A. baumannii* bacteremia, and the results showed that both of the two scoring systems were good independent prognostic factors. SOFA score > 8 and APACHE II > 29 were positively correlated with 14-day mortality, while compared with APACHE II score, SOFA score calculation is simpler and more practical, which can be used for early identification of clinical high-risk patients. Our study observed a very good correlation between APACHE II and SOFA scoring systems (Figure 7) with Pearson’s rho correlation coefficient of (*r*^2^ = 0.4410, *P* < 0.001), and showed that SOFA and APACHE II scores determined at the onset of *A. baumannii* bacteremia are reliable predictors of mortality. Though the use of serial SOFA scores would seem to provide a more effective representation of the dynamics of illness (Ferreira et al., 2001), such calculations can be very laborious. Our study showed that SOFA and APACHE II assessed at the onset of *A. baumannii* bacteremia is effective for the prediction of mortality. Use of a one-time scoring system is obviously much easier for practicing clinicians.

This study has the following limitations: (1) since this study was a retrospective study, it was not possible to determine whether the death was caused by *A. Baumannii* bacteremia, so the attributable mortality could not be analyzed; (2) since the included cases were single-center samples, the findings of this study cannot be generalized to other regions; (3) this study is prone to bias because of its retrospective characteristic; (4) because of the small sample size, the research results and conclusions are only for reference. We suggest that future studies from different institutes and different geographic areas evaluate the efficacy of these scoring systems in predicting mortality from *A. baumannii* bacteremia.

## Conclusion

The antibiotic resistance rate of *A. baumannii* bacteremia is high, and the incidence is highest in the ICU. Carbapenem-resistant *A.baumannii* (CRAB) was found in 77 cases (70%), MDR *A.baumannii* (in 76 cases (69.1%) and 3 cases (2.7%) were pan drug resistant (PDR). There was a significant difference in survival time between MDR group and non-MDR group. Colistin was the most sensitive to *A. baumannii*, but the utilization rate was relatively low. The SOFA score and APACHE II score can reflect the severity of *A.baumannii* bacteremia patients and evaluate the 28-day prognosis, which is helpful for early diagnosis and timely formulation of a treatment plan, and has important guiding significance for reducing mortality. In addition, for the convenience of calculation, SOFA score may have clinical application value in predicting the mortality of *A.baumannii* bacteremia.

## Data availability statement

The original contributions presented in this study are included in the article/supplementary material, further inquiries can be directed to the corresponding author/s.

## Ethics statement

The studies involving human participants were reviewed and approved by the Tianjin Medical University General Hospital’s Ethics Committee (NO. IRB2022-WZ-077). The patients/participants provided their written informed consent to participate in this study.

## Author contributions

YZ and LinZ acquired the data. LinaZ and YC revised the manuscript, worked on the English, and made the final version. All authors contributed to the article and approved the submitted version.

## Abbreviations

ICU: intensive care unit
APACHE II: Acute Physiology and Chronic Health Evaluation II
SOFA: Sequential Organ Failure Assessment
GCS: Glasgow coma scale
CRAB: Carbapenem-resistant *A.baumannii*
CSAB: Carbapenem-susceptible *A.baumannii*
MDR: Multidrug-resistant
XDR: extensively drug resistant
PDR: pan drug resistant
ROC: Receiver operating characteristic curve
MODS: Multiple Organ Dysfunction Syndrome
CRRT: Continuous Renal Replacement Therapy
CLSI: Clinical and Laboratory Standards Institute
EUCAST: European Committee on Antimicrobial Susceptibility Testing
HIS: health information system
LIS: Laboratory information system
ALT: alanine aminotransferase
AST: aspartate aminotransferase
ALB: albumin.
